# Supplementary material for: High Overexpression of SiAAP9 Leads to Growth Inhibition and Protein Ectopic Localization in Transgenic Arabidopsis
Source: Int J Mol Sci. 2024 May 27;25(11):5840. doi: 10.3390/ijms25115840 (PMC11172308; doi:10.3390/ijms25115840)
Supplement: Supplementary file 1 [file ijms-25-05840-s001.zip › Supplementary Figures.pdf]

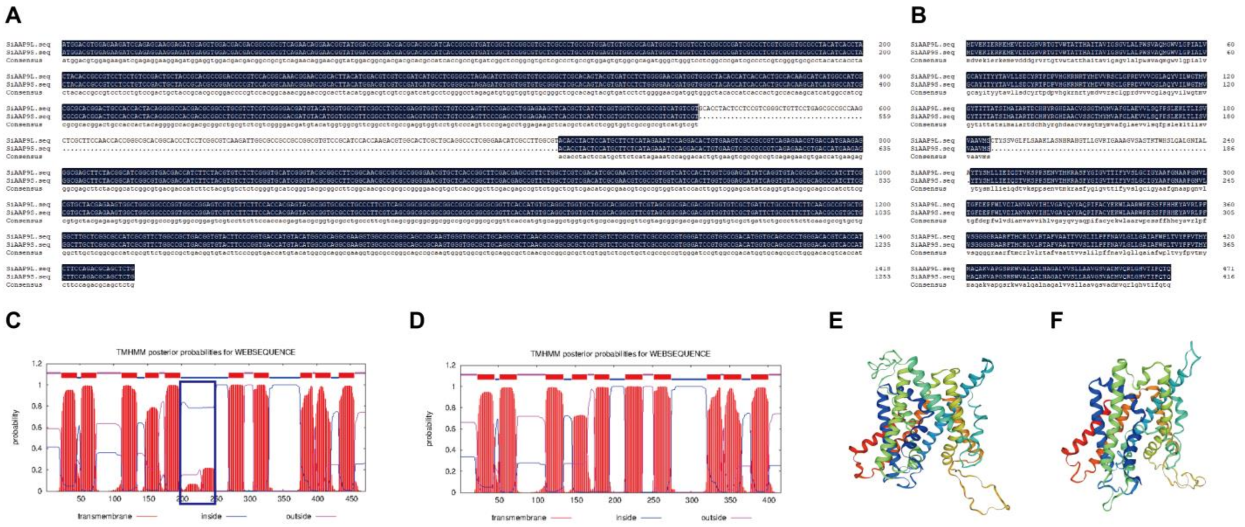

Figure S1. Sequence alignment and coding protein analysis of two alternative splicing events.

(A) DNA sequence alignment of two transcripts.

(B) Sequence alignment of amino acids encoded by *SiAAP9L* and *SiAAP9S*.

(C, D) Coding sequences of *SiAAP9L* and *SiAAP9S* are subjected to the TMHMM-2.0 website for transmembrane domain prediction.

(E, F) Swiss Model website for predicting the protein tertiary structure of the SiAAP9L and SiAAP9S.

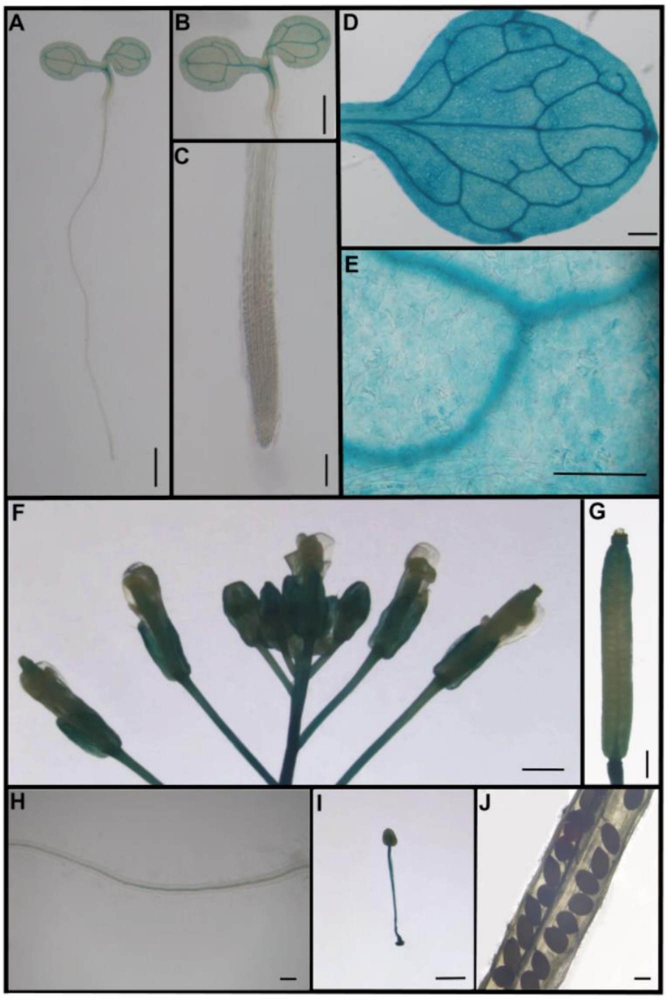

Figure S2. *SiAAP9* is widely expressed in *Arabidopsis* by GUS staining analysis.

Representative images from 20 individual transgenic lines expressing *SiAAP9 pro:GUS* are shown: 4-DAG seedling **(A)**, magnifications of the cotyledons **(B)** and root tips **(C)** of 4-DAG seedling, leaf from 21-DAG plants **(D)**, epidermal cells **(E)**, inflorescence **(F)**, mature pistil **(G)**, root **(H)**, developing anther **(I)**, developing seed **(J)**. Bars = 2 mm for **(A)**, **(D)**, and **(F)**, 100 μm for **(B)**, **(C)**, **(E)**, **(G)**, **(H)**, **(I)** and **(J)**.

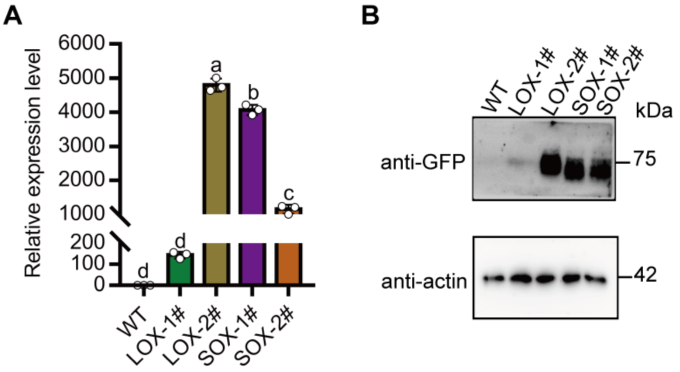

Figure S3. Quantification of the expression levels in WT and *SiAAP9-OX* transgenic *Arabidopsis* lines that were grown on 1/2 MS medium.

(A, B) Detection of expression levels of WT, *LOX-1#*, *LOX-2#*, *SOX-1#* and *SOX-2#* through RT-qPCR and Western Blot. Data represent means ± SD (n=3). Significant difference is analyzed based on one-way ANOVA by Duncan test, lowercase letters at $p < 0.05$.

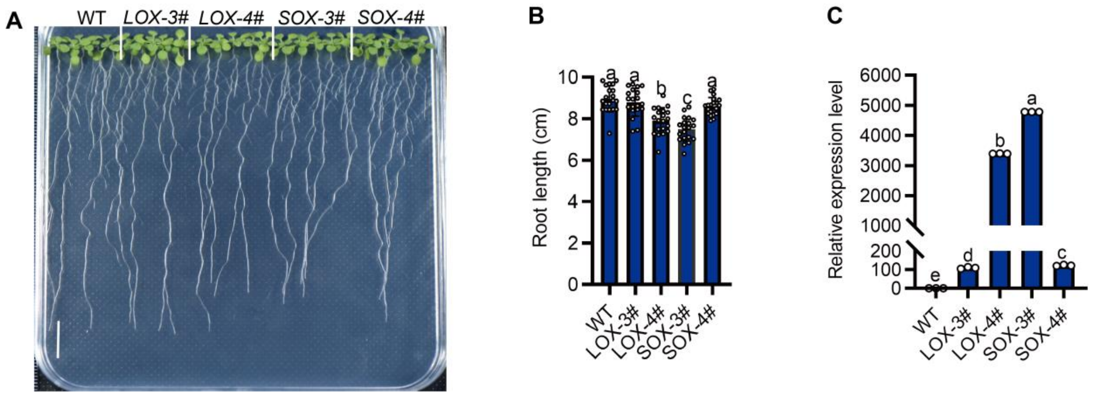

Figure S4. *SiAAP9* inhibits the growth and development of primary roots.

(A) Phenotypes of WT and *SiAAP9-OX* transgenic *Arabidopsis* lines (*LOX-3#*, *LOX-4#*, *SOX-3#*, *SOX-4#*) grown on 1/2 MS medium for 2 weeks. Scale bar = 1 cm.

(B) Primary root length was measured of WT and *SiAAP9-OX* transgenic *Arabidopsis* lines (*LOX-3#*, *LOX-4#*, *SOX-3#*, *SOX-4#*) grown on 1/2 MS medium for 2 weeks (n=20).

(C) Detection of *SiAAP9* expression levels of WT, *LOX-3#*, *LOX-4#*, *SOX-3#*, *SOX-4#* through RT-qPCR.

(B-C) Significant difference is analyzed based on one-way ANOVA by Duncan test, lowercase letters at $p < 0.05$.

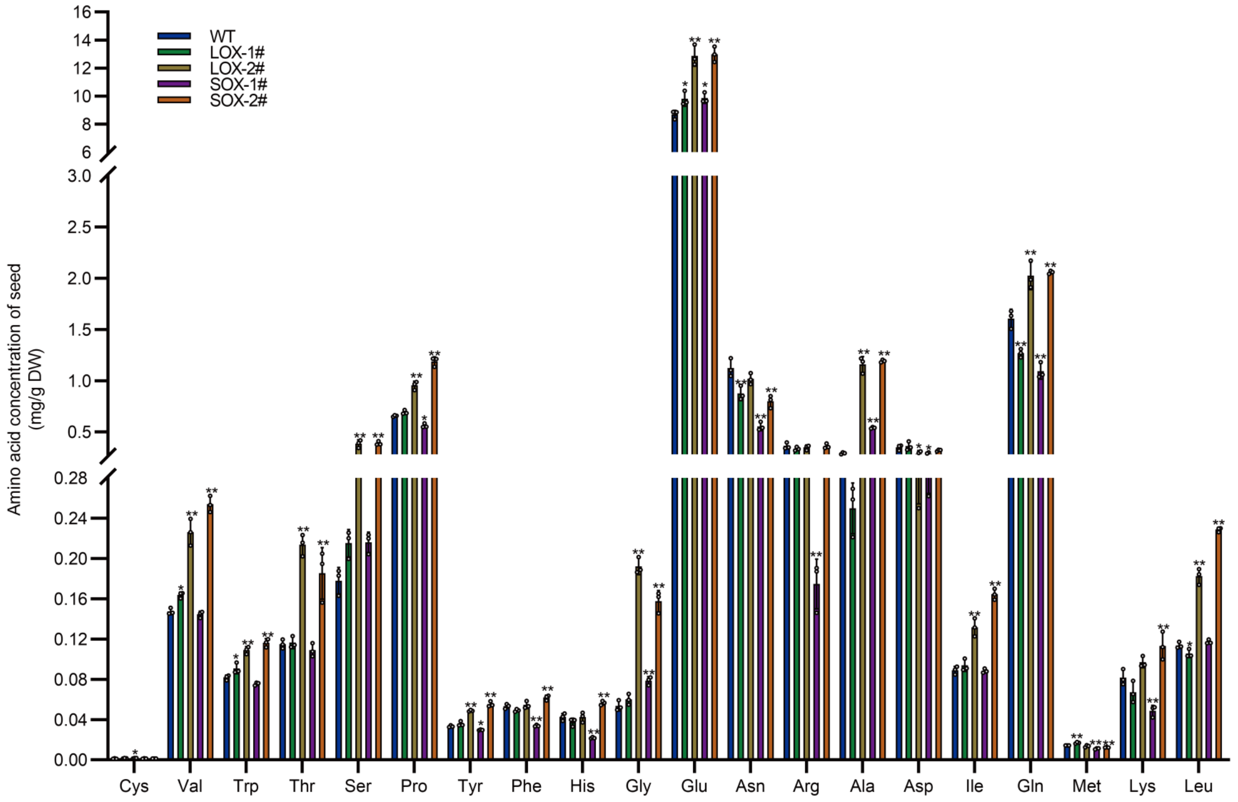

Figure S5. Amino acid concentration of seeds at mature stage in WT, *LOX-1#*, *LOX-2#*, *SOX-1#* and *SOX-2#* lines. Data represent the mean ± SD (n=3). Asterisks indicate significant differences based on one-way ANOVA followed by Duncan test: * $p < 0.05$, ** $p < 0.01$.

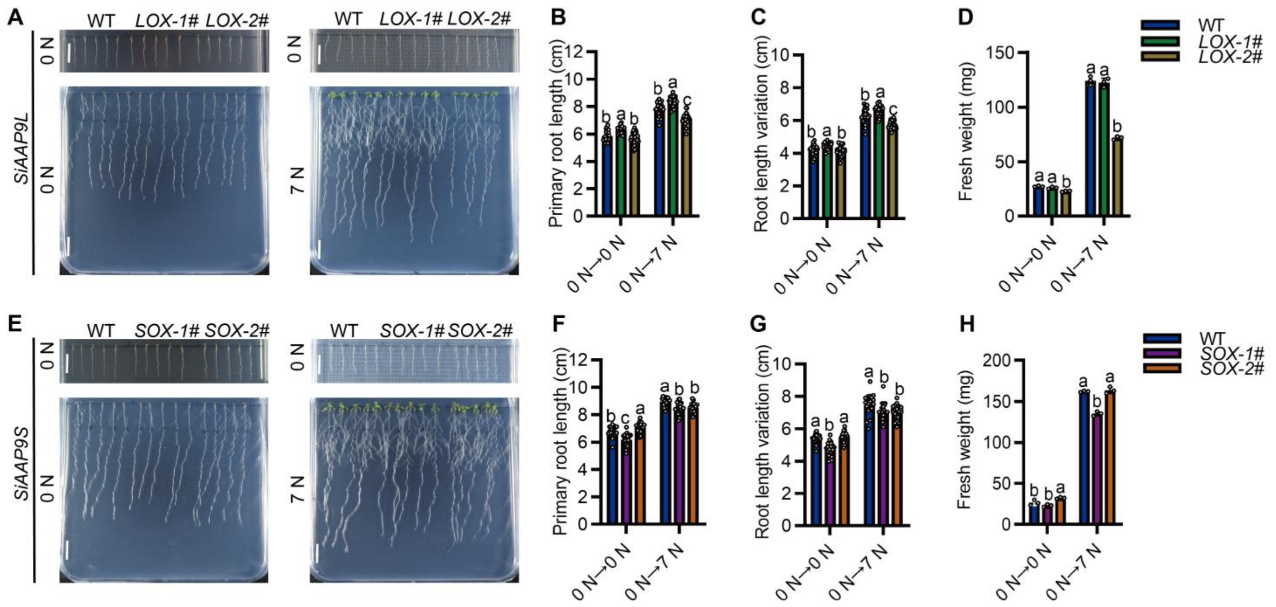

Figure S6. The phenotype analysis of WT and *SiAAP9-OX* transgenic *Arabidopsis* lines under 7 mM NO_3^-.

(A, E) WT, *LOX-1#*, *LOX-2#*, *SOX-1#* and *SOX-2#* were vertically cultivated on 0 mM NO_3^- for 4 days (upper panels), then were transferred to 7 mM NO_3^- and allowed to grow for an additional 8 days (lower panels). The experiments were biologically repeated at least three times, yielding consistent results. Scale bar = 1 cm.

(B, F) Primary root length was measured in WT and *SiAAP9-OX* transgenic *Arabidopsis* lines at 12 days. Data represent the mean ± SD (n = 20).

(C, G) Primary root length was measured in WT and *SiAAP9-OX* transgenic *Arabidopsis* lines after transferring the plate at 8 days. Data represent the mean ± SD (n = 20).

(D, H) Fresh weight was measured in WT and *SiAAP9-OX* transgenic *Arabidopsis* lines at 12 days. Data represent the mean ± SD (n = 20).

(B-D, F-H) Significant difference is analyzed based on one-way ANOVA by Duncan test, lowercase letters at $p < 0.05$.

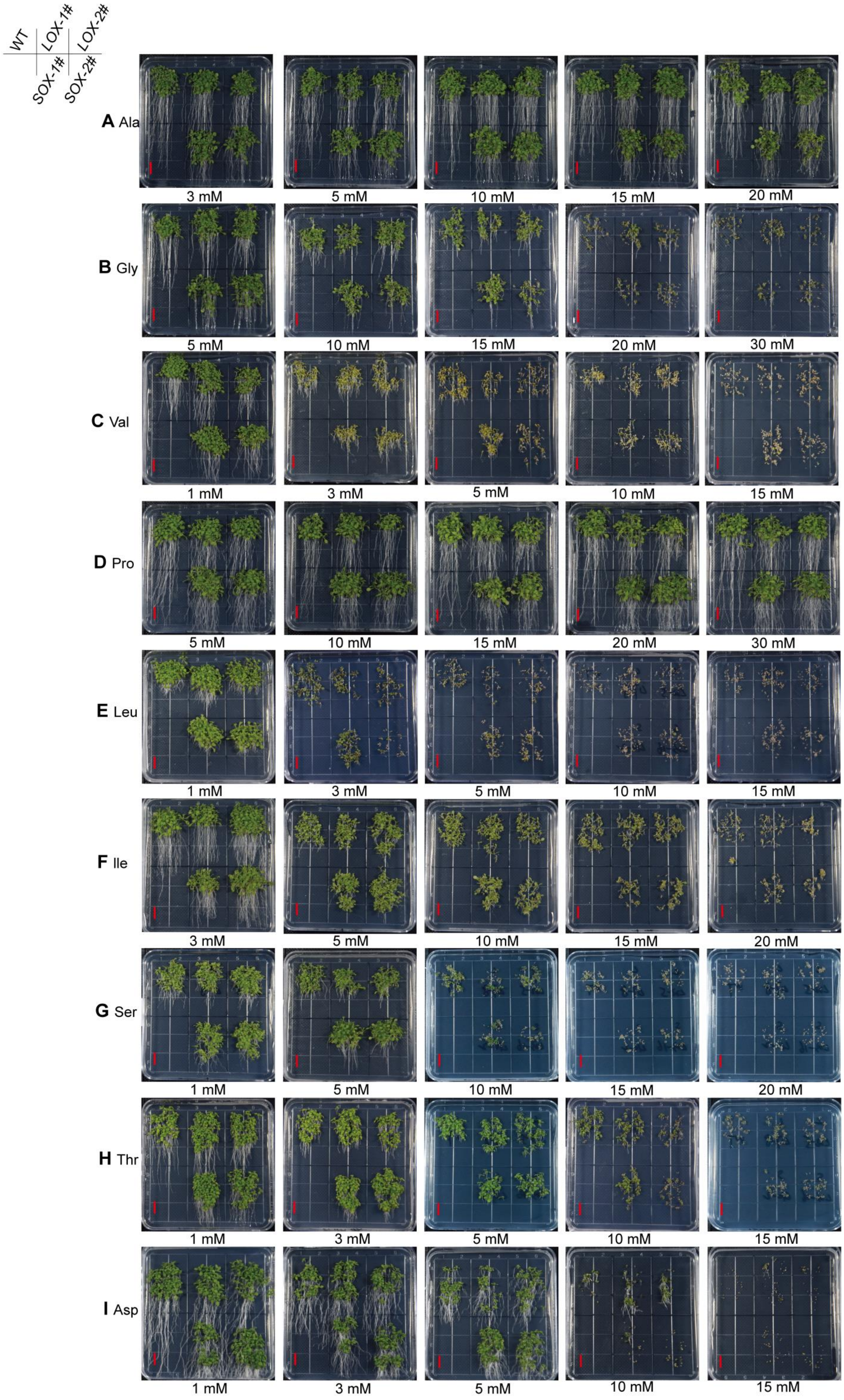

WT
LOX-1#
LOX-2#
SOX-1#
SOX-2#
A Ala
3 mM
5 mM
10 mM
15 mM
20 mM
B Gly
5 mM
10 mM
15 mM
20 mM
30 mM
C Val
1 mM
3 mM
5 mM
10 mM
15 mM
D Pro
5 mM
10 mM
15 mM
20 mM
30 mM
E Leu
1 mM
3 mM
5 mM
10 mM
15 mM
F Ile
3 mM
5 mM
10 mM
15 mM
20 mM
G Ser
1 mM
5 mM
10 mM
15 mM
20 mM
H Thr
1 mM
3 mM
5 mM
10 mM
15 mM
I Asp
1 mM
3 mM
5 mM
10 mM
15 mM

Figure S7. *SiAAP9-OX* transgenic *Arabidopsis* lines showed no significant response to nine amino acids, compared with WT. Nine amino acids are Ala (**A**), Gly (**B**), Val (**C**), Pro (**D**), Leu (**E**), Ile (**F**), Ser (**G**), Thr (**H**) and Asp (**I**). Scale bar = 1 cm.

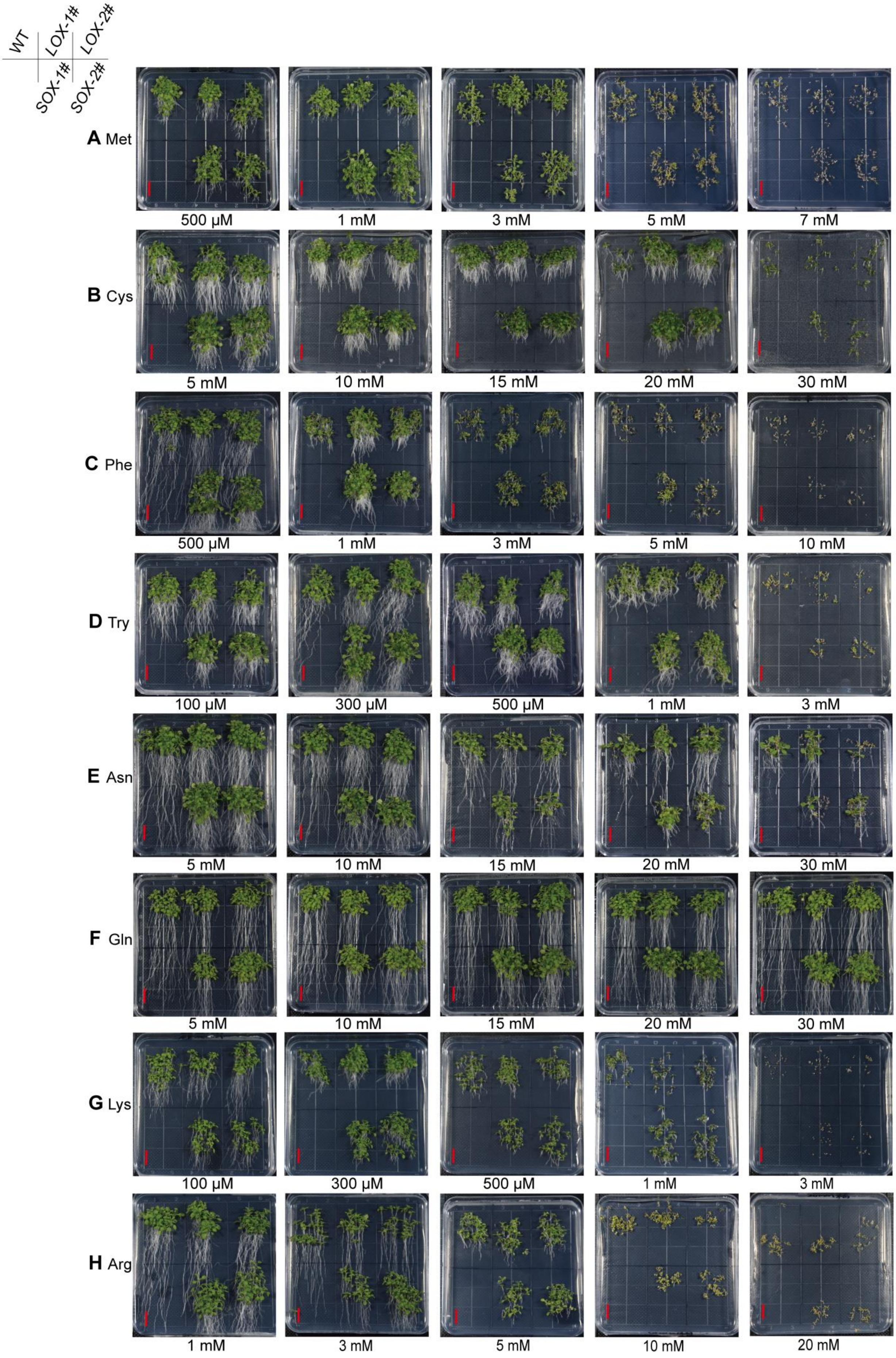

Figure S8. *SiAAP9-OX* transgenic *Arabidopsis* lines showed no significant response to eight amino acids, compared with WT. Eight amino acids are Met (**A**), Cys (**B**), Phe (**C**), Try (**D**), Asn (**E**), Gln (**F**), Lys (**G**) and Arg (**H**). Scale bar = 1 cm.

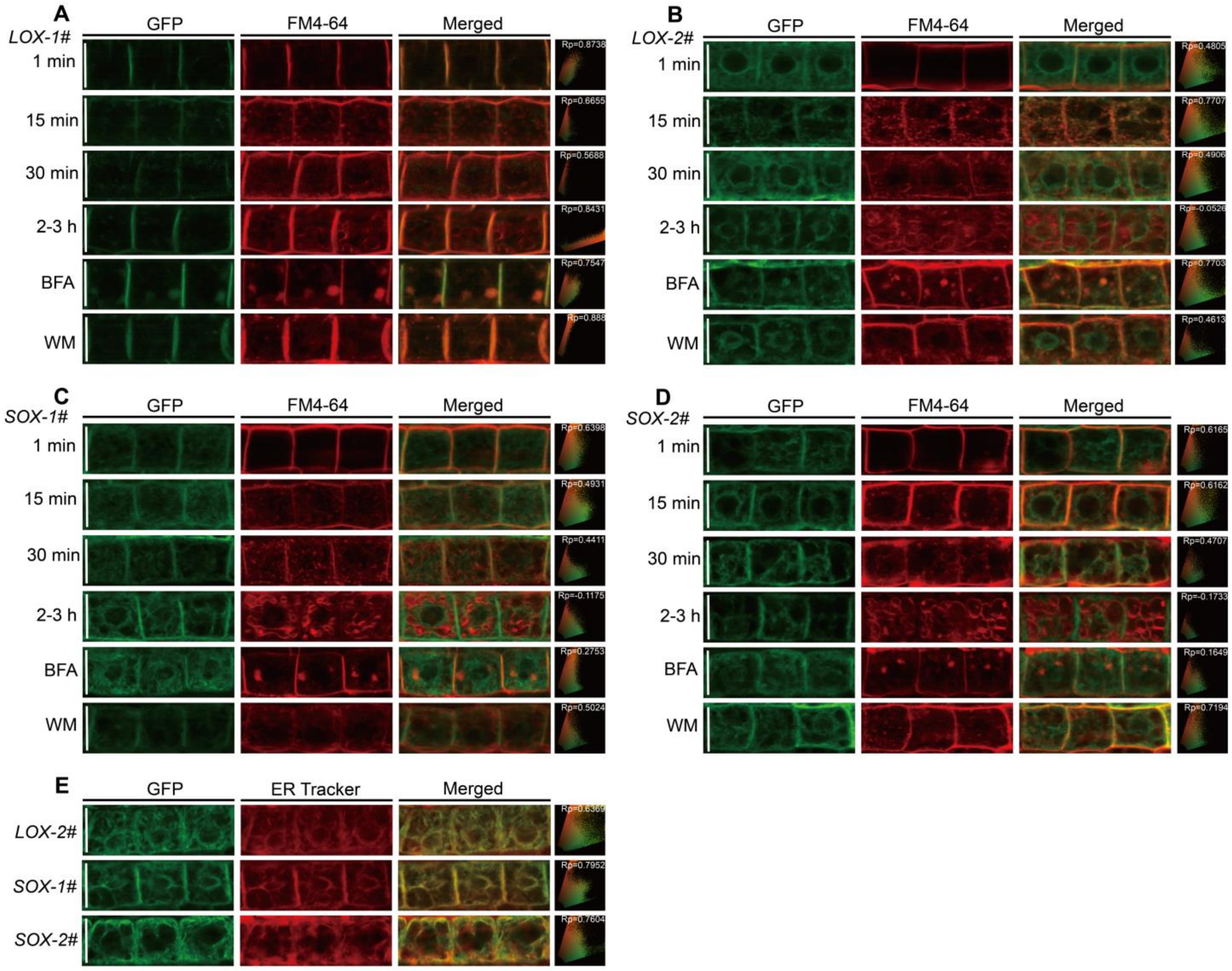

Figure S9. Subcellular localization of SiAAP9L and SiAAP9S in *Arabidopsis* were pulse labeled with FM4-64.

(A-D) Roots of four *SiAAP9-OX* transgenic *Arabidopsis* lines grown on 1/2 MS medium for 4 days were pulse labeled with FM4-64 for 1 min, 15 min, 30 min or for 2-3 h and followed by BFA treatment for 50 min or WM treatment for 1 h, respectively.

(E) Seedlings of *LOX-2#*, *SOX-1#* and *SOX-2#* grown on 1/2 MS medium for 4 days were stained with ER Tracker for 1 hour.

(A-E) Each image shows the corresponding Pearson correlation coefficient (Rp). Bar = 20 μm.
